# Jasmonic Acid Signals Involved in *Valsa* Canker Resistance Caused by C2H2-Type Transcription Factor PbeSTOP2 in *Pyrus betulifolia*

**DOI:** 10.3390/cimb48010014

**Published:** 2025-12-23

**Authors:** Longgang Zuo, Zhihong Liu, E Sun, Yuan Lu, Minrui Cai, Hongqiang Yu, Junying Zhao, Cunwu Zuo

**Affiliations:** 1 Gansu Academy of Forestry Sciences, Lanzhou 730020, China; zlg11282025@163.com; 2 College of Horticulture, Gansu Agricultural University, Lanzhou 730070, China; 18189609278@163.com (Z.L.); se107332002se@126.com (E.S.); ly16380@126.com (Y.L.); caimrlz@126.com (M.C.); yu_destiny@126.com (H.Y.); 3 Key Laboratory of Crop Science in Arid Environment of Gansu Province, Lanzhou 730070, China

**Keywords:** *Valsa* canker, transcription factor, disease resistance, hormonal signals, immune responses

## Abstract

*Valsa* canker, a destructive necrotrophic disease caused by *Valsa* species (*V. mali* and *V. pyri*), threatens global apple and pear production. Resistance breeding has gained much attention because of its environmental friendliness and effectiveness, making it extremely critical to obtain key disease resistance genes. In this study, we identified that the expression of *PbeSTOP2,* a C2H2-type transcription factor in ‘Duli-G03’ (*Pyrus betulifolia*, rootstock of pear), was induced in response to signals from *Vp* and *Vp* metabolites (*Vp*M). Transient expression in fruits and stable overexpression in suspension cells demonstrated that *PbeSTOP2* enhances resistance to *Vp*. In overexpressing cells, genes associated with jasmonic acid (JA) and pattern-triggered immunity (PTI) signaling were upregulated, and endogenous JA and auxin (IAA) levels increased. These results revealed that JA signaling was involved in the enhanced *Valsa* canker resistance conferred by *PbeSTOP2* overexpression. This finding on host disease resistance should facilitate the breeding efforts for *Valsa* canker resistance.

## 1. Introduction

*Valsa* canker, a destructive disease caused by necrotrophic fungi (*Valsa mali* and *V. pyri*), poses a significant threat to apple and pear production [1]. The pathogen mainly attacks the bough and lateral branches via wounds, ultimately reducing orchard yield and fruit quality. Although chemical fungicides are widely used for control, their application is constrained by potential environmental and health risks. Resistance breeding, an environmentally friendly and effective practice, is commonly approved but has been restricted due to a prolonged breeding cycle [2]. Considering the multiple advantages of advanced molecular breeding, it is urgent to identify key genes regulating *Valsa* canker resistance from highly resistant germplasm.

During the long-term progress of struggling against pathogenic microbes, plants have evolved a natural two-tiered immune system, defined as horizontal resistance and vertical resistance [3]. First, pattern recognition receptors (PRRs) on the plant cell membrane could sense the signals from pathogens that produce harmful signaling molecules with special conserved motifs and trigger horizontal resistance, known as PRR-triggered immunity (PTI) [4]. Certain pathogens can inject virulence factors into plant cells to repress the activation of PTI for successful infection. After breaching the cell-surface defenses controlled by PRRs, these effector proteins can be specifically recognized by intracellular nucleotide-binding, leucine-rich repeat receptors (NLRs). This recognition in turn activates a stronger, vertical resistance response known as effector-triggered immunity (ETI) [4]. Subsequently, the local signals are transmitted throughout the plant and activate systematic acquired resistance (SAR). A series of signals, such as callus deposition, stomatal closure, hypersensitive response (HR), accumulation of reactive oxygen species (ROS), salicylic acid (SA) and jasmonic acid (JA), and transcription factors (TFs), take the crucial roles on initiation and signal transduction of immune responses [5,6]. Notably, the JA pathway is pivotal across multiple immune stages. Pathogen infection can disrupt the synthesis, metabolism, and signal transduction of JA within the host. The increase in JA levels activates downstream defense genes, thereby enhancing resistance to necrotic pathogens. For instance, exogenous application of methyl jasmonate (MeJA) can significantly enhance resistance against *Sclerotinia sclerotiorum* in both oilseed rape (*Brassica napus*) and soybean (*Glycine max*).

TFs are protein molecules that mediate intracellular signals and stress responses via regulating gene transcription [7,8]. Numerous TF families, such as zinc finger protein (ZFP), basic–leucine zipper (bZIP), myeloblastosis (MYB), N-acetylcysteine (NAC), high-mobility group (HMG), and APETALA2/ethylene-responsive element binding factor (AP2/ERF), have been shown to play vital and unique roles in plant specificity regulation [9]. Among these, ZFPs are the most widely distributed proteins in eukaryotic genomes, which can be classified into C2H2, C8, C6, C3HC4, C2HC, LIM, C4, C3H, and C4HC3 based on the number and position of cysteine (Cys) and histidine (His) residues [10,11]. C2H2 contains a zinc finger structure consisting of approximately 30 amino acids, with a conserved sequence of X2-Cys-X(2-4)-Cys-X12-His-X(3-5)-His (X represents any amino acid). The first plant-specific C2H2 member, *ZPT2-1* (EPF1), was isolated in *Petunia hybrida* [12]. After that, a large number of C2H2-type ZFPs were successfully identified from various organisms, such as *Arabidopsis thaliana* [10], *Oryza sative* [13], *Triticum* [14], *Solanum tuberosum* [15], *Solanum lycopersicum* [16], and *Nicotiana tabacum* [17], with the member size recorded as 176, 189, 122, 79, 118 and 211, respectively. In-depth investigations revealed the crucial roles of numerous C2H2-type ZFPs in plant immunity. For instance, overexpression of the pepper C2H2-type ZFP gene *CAZFP1* in transgenic *Arabidopsis* plants can enhance resistance to infection by *Pseudomonas syringae* [18]. In tomatoes, *SlZF3* regulates plant height by directly inhibiting genes in the gibberellin biosynthesis pathway [19]. In addition, the C2H2-type ZFP transcription factor *MdZAT10* in apples can significantly accelerate leaf senescence by promoting the expression of aging-related genes [20].

Duli is a rootstock belonging to *Pyrus betulifolia* that is widely planted in northern China as a rootstock due to its superior characteristics, such as wide adaptability and good graft compatibility. It has been proven to have high resistance against the attack of various pathogenic microbes, including the pathogen of *Valsa* canker [21]. ‘Duli-G03’ is a solitary plant identified by our research team during field investigations, which exhibits exceptional resistance to *Valsa* canker. Multiple previous investigations have been focused on the resistance mechanism of Duli against *V. pyri* infection. For instance, the *PbeKCS1/10-1*, *PbeMPK4-1*, *MdCN19*, and *MdCN11* genes are determined as crucial signals that are involved in the responses of ‘Duli-G03’ (*P. pyrifolia*) against *V. pyri* [22,23]. In addition, based on the gene family analysis and functional validation, receptor-like proteins *PbeRP23* and *PbeRP27* are screened, which are key regulators for the *Valsa* canker resistance of ‘Duli-G03’ [24]. Unfortunately, whether C2H2 members also take vital roles in *Valsa* canker resistance has not been elucidated.

In a preliminary analysis, the expression of *PbeSTOP2* was significantly activated when ‘Duli-G03’ suspension cells were challenged with signals of *Valsa* canker. In this study, by means of transient expression and stable transformation, we determined that *PbeSTOP2* positively conferred *Valsa* canker resistance of ‘Duli-G03’ cells and apple and pear fruits. Moreover, the mechanistic investigations revealed that JA signals are involved in the resistance launching of immune response derived from *PbeSTOP2*.

## 2. Materials and Methods

### 2.1. Plant Materials and Pathogen Isolation

Calli was induced from leaves of pear ‘Duli-G03’ (*Pyrus betulifolia*) tissue-cultured plantlets. Vigorous callus was then subcultured in liquid MS medium and shaken at 130 rpm in darkness at 25 °C. After a week, cells were collected by filtration through a 40-mesh sieve. These suspension cells were maintained in MS medium under controlled conditions (25 °C, 130 rpm). The pathogenic fungus *Valsa pyri* strain *Vp*-001 was cultivated on potato dextrose agar (PDA) in complete darkness at 25 °C [25,26]. For *Valsa pyri* metabolites (*Vp*M) extraction, static cultures of *Vp*-001 were grown in potato dextrose broth (PDB) at 25 °C without light. After 72 h of incubation, the culture supernatant was collected via centrifugation (8000× *g*, 10 min) and designated as undiluted *Vp*M (100% concentration).

### 2.2. Bioinformatic Analysis of PbeSTOP2

The coding sequence (CDS) and corresponding protein sequence of *PbeSTOP2* (Chr3.g19672.m1) were acquired from the publicly available Genome Database for *Rosaceae* (GDR: https://www.rosaceae.org/species/malus/all, accessed on 8 May 2025). Domain architecture prediction for the protein encoded by *PbeSTOP2* was carried out with the web-based program SMART (http://smart.emblheidelberg.de/, accessed on 8 May 2025), using a significance threshold of E-value ≤ 1 × 10^−5^. Physicochemical characteristics and subcellular localization were evaluated, respectively, through ExPASy ProtParam (https://web.expasy.org/protparam/, accessed on 9 May 2025) and WoLF PSORT (https://www.genscript.com/wolf-psort.html, accessed on 9 May 2025). Candidate gene sequences from *Arabidopsis thaliana*, *Oryza sativa*, and *Solanum lycopersicum* were sourced from the *Arabidopsis* Information Resource (TAIR, https://www.arabidopsis.org/, accessed on 10 May 2025) and the National Center for Biotechnology Information (NCBI, https://www.ncbi.nlm.nih.gov/, accessed on 10 May 2025). Multiple sequence alignment was executed in Clustalx 2.0, and resultant alignments were subsequently analyzed with MEGA 8.0 (http://www.megasoftware.net, accessed on 11 May 2025) for evolutionary inference. A phylogenetic tree was generated via the neighbor-joining approach under the following settings: P-distance substitution model, pairwise deletion treatment, and 1000 bootstrap replications. The 2000 bp promoter region upstream of the *PbeSTOP2* initiation site was extracted from the same database, and *cis*-regulatory elements within this sequence were predicted using Plant CARE (https://bioinformatics.psb.ugent.be/webtools/plantcare/html/, accessed on 11 May 2025).

### 2.3. Subcellular Localization of PbeSTOP2

The full-length coding region of *PbeSTOP2* was ligated into the pCAMBIA1300-GFP vector. Following sequence verification, the fusion construct *35S:PbeSTOP2-GFP* was introduced into *Agrobacterium tumefaciens* strain GV3101 [27]. *35S:PbeSTOP2-GFP* was activated, resuspended, and pressure-infiltrated into the leaves of *Nicotiana benthamiana*, with empty vector 35S: GFP as the control. After 48 h dark incubation, infiltrated leaf tissues were co-stained with 4′,6-diamidino-2-phenylindole (DAPI, 1 μg/mL) for nuclear visualization. Subcellular localization analysis was conducted on a laser scanning confocal microscope (FV1200, Olympus Corporation, Tokyo, Japan.).

### 2.4. Exogenous Hormone Treatment

For exogenous hormonal treatments, methyl jasmonate (MeJA) or SA at a concentration of 50 μM was added to ‘Duli-G03’ cell suspensions and incubated at 25 °C in the darkness. SA was purchased from Shanghai Yuan Ye Biotechnology Co. (Shanghai, China), and MeJA was purchased from Sigma-Aldrich ((St. Louis, USA). Following collection of cells at designated intervals (0, 1, 3, 6 h post-treatment), total RNA was isolated for subsequent qRT-PCR analysis performed in accordance with Zhao et al. [21].

### 2.5. Transient Expression of PbeSTOP2 in Fruits

The full-length coding sequence of PbeSTOP2 was cloned into the pFGC5941 vector via homologous recombination, and the recombinant plasmid PFGC5941-*PbeSTOP2* was obtained. The genome-specific fragment *PbeSTOP2* (318 bp) was introduced into the pTRV2 vector. All constructs were transformed into *Agrobacterium* strain GV3101. Positive colonies of GV3101 harboring PFGC5941-*PbeSTOP2*, pTRV2-*PbeSTOP2* and empty vectors were cultured in 15 mL LB liquid medium containing 50 mg/L kanamycin and 50 mg/L gentamicin, shaking (180 rpm) at 28 °C to OD_600_ value of 1.0, followed by centrifugation at 5000 rpm for 8 min. For functional validation, transient overexpression and Virus-Induced Gene Silencing (VIGS) assays were conducted on ‘Huangguan’ pear and ‘Yanfu 6’ apple fruits via *Agrobacterium*-mediated infiltration [22]. *Agrobacterium* containing an empty vector was injected as the control. Three days post-infiltration, *Vp* was inoculated at the infiltration sites. Samples were incubated in darkness at 25 °C, and lesion diameters were measured every 12 h using digital vernier calipers.

### 2.6. Stable Overexpression and Functional Verification of PbeSTOP2

Suspension cells derived from ‘Duli-G03’ were pre-cultured in a constant-temperature oscillator at 25 °C for a duration of 3 days. *Agrobacterium tumefaciens* strain GV3101 carrying the 35 S: *PbeSTOP2* recombinant plasmid was cultured until the bacterial culture reached an OD_600_ of 1.0, followed by resuspension in 2-(4-morpholino)ethanesulfonic acid (MES) medium. Afterwards, 2 mL of the prepared *Agrobacterium* suspension was mixed with 20 mL of the pre-cultured ‘Duli-G03’ suspension cells, and the mixture was incubated for 20 min. After removing residual *Agrobacterium* by centrifugation, treated cells were incubated for 3 days. Residual bacteria were eliminated by antibiotic treatment, and cells were plated on MS solid medium for 20-day dark incubation at 25 °C [22]. Overexpression lines were selected based on PCR and Quantitative real-time PCR (qRT-PCR) validation. The transgenic and wild-type (WT) cells were maintained separately in MS liquid medium (25 °C, 130 rpm), then 2 mL were transferred to MS solid medium. After 3 days of incubation in darkness at 25 °C, each plate was inoculated with a 5 mm plug of *Vp* strain. Lesion diameters were measured every 12 h using digital vernier calipers [22]. *PbeSTOP2*-overexpressing cells were infected with *Vp*M at a concentration of 20%. For the control, the WT cells were treated under the same conditions described above. All samples were harvested at 0, 1, 3, and 6 h post-treatment, and cell viability was assessed via the methyl thiazolyl tetrazolium (MTT) assay [24]. For ROS detection, suspension cells of the *PbeSTOP2*-overexpressing cells and the WT were treated with 20% *Vp*M solution. Samples were collected at 0, 1, 3, and 6 h and incubated with the ROS-specific fluorescent dye H2DCFDA (10 µmol L^−1^) for 10 min at 37 °C in the dark, followed by quantification in labeled cells using a Spark microplate reader (Tecan Group Ltd., Männedorf, Switzerland). The excitation and emission wavelengths were 488 nm and 525 nm, respectively.

### 2.7. Gene Expression Assays

The expressional patterns were detected by extracting data from released RNA-seq data and using a Quantitative real-time PCR (qRT-PCR) assay. For the RNA-seq data, ‘Duli-G03’ suspension cells treated with *Vp*M for 1, 3, and 6 h were utilized [21]. Primers for both target were designed with the online tool Primer 3.0 plus (https://www.primer3plus.com/index.html, accessed on 15 May 2025) (Table 1). The reference gene *TUB* (β-Tubulin, Chr3.g20215) in *Pyrus* spp. was identified based on the method by Pessina et al. [28]. Total RNA was extracted from suspension cells of ‘Duli-G03’ using the Tiandz RNA extraction kit (Catalog No. 71203-50, Tiangen Biotech, Beijing, China) following the manufacturer’s protocol. The extracted RNA was treated with DNase I (1 U/μg RNA) at 37 °C for 30 min to eliminate genomic DNA contamination. First-strand cDNA was then synthesized from 1 μg of the DNase-treated RNA using PrimeScript™ Reverse Transcriptase (Takara Bio Inc., Kusatsu, Japan) and oligo(dT)_18_ primers in a 20-μL reaction volume. The reverse transcription was performed at 42 °C for 30 min, with subsequent enzyme inactivation at 85 °C for 5 s. qPCR was performed with SYBR^®^ Green Premix (Bio-Rad) on a CFX96 Touch™ system. Program: 95 °C for 30 s; 40 cycles of [95 °C for 5 s, 60 °C for 30 s, 72 °C for 30 s]; melting curve from 65 °C to 95 °C at 0.5 °C/5 s. Efficiency was determined via a standard curve from 10-fold cDNA dilutions, calculated as E = (10^−1/slope^ − 1) × 100%. All primer efficiencies were 90–110% (R^2^ > 0.99). Expression was normalized to *TUB* and calculated using the 2^−ΔΔCT^ − method [29]. Statistical analysis was performed in Microsoft Excel 2010. All experiments included at least three biological and technical replicates.

### 2.8. Endogenous Hormone Assays

Liquid chromatography-tandem mass spectrometry (LC-MS) for the determination of endogenous cellular hormones [30]. First, cell samples (1 g) were collected, ground in liquid nitrogen, and placed in 15 mL centrifuge tubes. Then, 10 mL of pre-cooled methanol-formic acid solution (99:1) was added, sonicated at 22 °C for 2 min, and stored at 4 °C for 12 h. Next, the extract was centrifuged at 10,000 r/min for 10 min, 1.0 mL of supernatant was aspirated, and H_2_O was added to the 10 mL of solution. Then, the ODS C18 solid phase extraction column was used for the adsorption of the upper samples, and the column was washed with 6 mL of 10% methanol solution in two batches. Finally, it was eluted with methanol-formic acid solution and fixed to 1.0 mL, and the sample was collected after filtration with a 0.22 μm microporous filter membrane. The instrument LC-MS coupled instrument (Agilent 1290-6460, Agilent Technologies, Santa Clara, CA, USA) was used for sample detection. LC conditions: Detection wavelength was set to 290 nm, column temperature maintained at 35 °C, and flow rate fixed at 0.3 mL/min. Mobile phase consisted of B (methanol): A (0.1% formic acid in water) with gradient elution programmed as follows: 0–1 min from 10% to 40% B, 1–2.5 min from 40% to 45% B, 2.5–5 min from 45% to 80% B, 5–5.1 min from 80% to 10% B, and 5.1–7.1 min held at 10% B; injection volume was 5 μL. MS conditions: Electrospray ionization source (ESI) was operated in negative ion mode with multiple reaction monitoring (MRM). Parameters included ion source temperature at 350 °C, drying gas flow rate at 11 L/min, nebulizer pressure at 35 psi, capillary voltage at 4 kV, and collision gas of high-purity nitrogen.

### 2.9. Statistical Analysis

The basic data were statistically analyzed in Microsoft Excel 2010 and visualized with Origin 2022. Differences between means were determined using Student’s *t*-test (* p < 0.05; ** p < 0.01).

## 3. Results

### 3.1. PbeSTOP2 Encoded a Typical C2H2-Type Zinc Finger Protein

**Figure 1:**
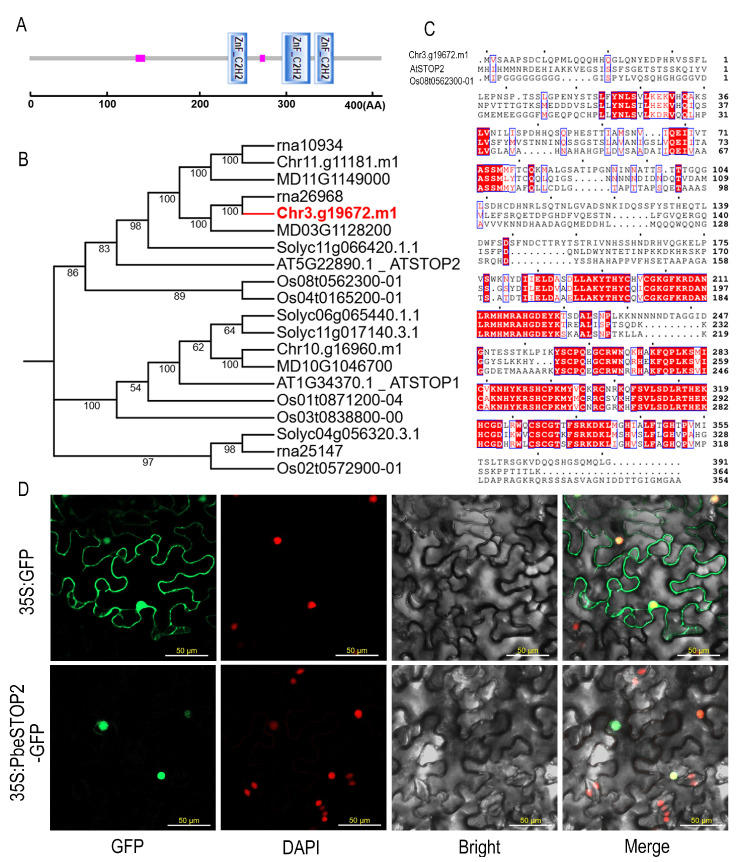
Bioinformatics characterization and subcellular localization of *PbeSTOP2*. (**A**) The domain architecture of PbeSTOP2 includes three characteristic C2H2-type zinc finger motifs. (**B**) Phylogenetic relationships between *PbeSTOP2* and homologous proteins from various species. (**C**) Multiple sequence alignment of *PbeSTOP2*, *ATSTOP2*, and *Os08t0562300-01*. (**D**) Cellular localization of PbeSTOP2 expressed in *N. benthamiana* leaves. Nuclei were stained with DAPI for reference.

Bioinformatics analysis of Chr3.g19672 was performed to obtain its related information. Domain prediction results demonstrated that the target gene encodes a protein that possesses three typical C2H2-type zinc fingers at 232 to 254, 295 to 328, and 333 to 355, respectively (Figure 1A). BLAST(NCBI BLAST+ 2.14.0, accessed on 11 May 2025) analysis indicated that the target protein shares high similarity with Sensitive To Proton Rhizo Toxicity (AtSTOP2) in *Arabidopsis*, leading to its designation as PbeSTOP2. Phylogenetic reconstruction revealed that *PbeSTOP2* exhibits closer evolutionary relationships with homologs from Rosaceae species compared to those from other plant families (Figure 1B). The results of the microcolinearity analyses indicate that *PbeSTOP2* is a direct ortholog of the *Arabidopsis thaliana AtSTOP2* and rice *Os08t0562300-01* (Figure S1). Based on sequence alignment, PbeSTOP2 shared 41.35% and 41.61% sequence identity with AtSTOP2 and Os08t0562300-01, respectively (Figure 1C). Additionally, transient expression in *Nicotiana benthamiana* leaves confirmed the nuclear localization of PbeSTOP2 (Figure 1D). Connectively, the above results indicated that PbeSTOP2 was a typical C2H2-type zinc finger protein and may possess unique functions in the nucleus.

### 3.2. PbeSTOP2 Responded to Vp and Related Defense Signals

**Figure 2:**
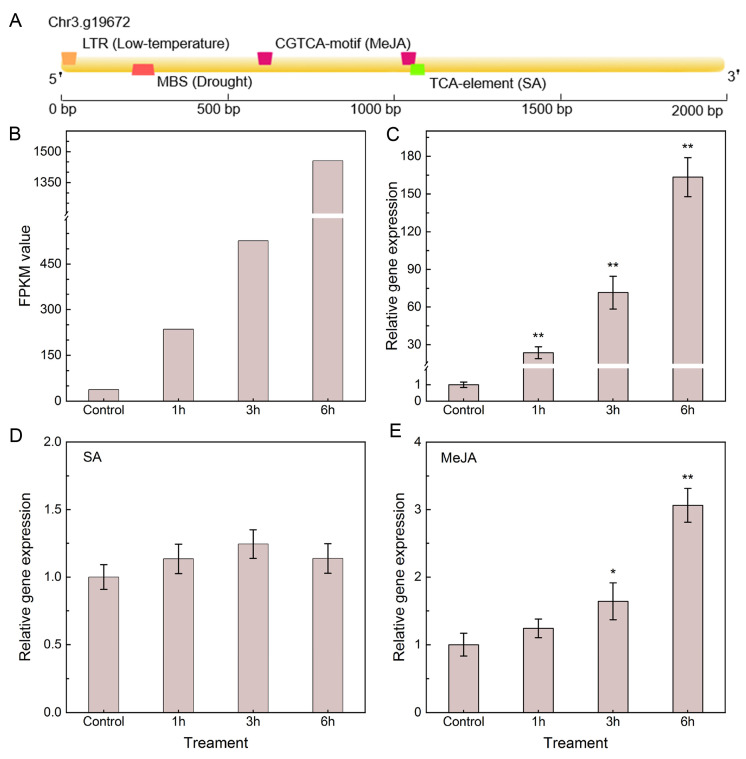
Prediction of *cis*-elements in the *PbeSTOP2* promoter and its expression profiles in ‘Duli-G03’ suspension cells following *Valsa pyri* metabolites (*Vp*M), MeJA, and SA treatments. (**A**) The promoter region of *PbeSTOP2* contained multiple stress response-related signaling components. (**B**) Transcript levels of *PbeSTOP2* based on RNA-seq data in ‘Duli-G03’ suspension cells exposed to 20% *Vp*M for 1, 3, and 6 h. (**C**) Transcript levels of *PbeSTOP2* using qRT-PCR validation of B. (**D**) Expression changes in PbeSTOP2 in response to exogenous SA application. (**E**) Expression profile of *PbeSTOP2* under exogenous MeJA treatment. Statistical annotations: * *p* < 0.05, ** *p* < 0.01 (*t*-test); bars show mean ± SD (*n* = 5).

We predicted the *cis*-element on the promoter region of *PbeSTOP2* to investigate its potential function. Multiple stress- and signaling-related *cis*-elements were identified, including those responsive to MeJA, SA, and various abiotic stresses (Figure 2A). We extracted RNA-seq data of *PbeSTOP2* after treating ‘Duli-G03’ suspension cells with *Vp*M. Compared with the control, the expression of *PbeSTOP2* was significantly up-regulated in ‘Duli-G03’ suspension cells during *Vp*M infiltration (Figure 2B). This expression pattern was further validated by qRT-PCR analyses (Figure 2C). We further monitored the response pattern of *PbeSTOP2* to exogenous SA and MeJA signaling in ‘Duli-G03’. The results showed that the expression of *PbeSTOP2* was not changed with exogenous SA exposure, but was robustly activated under MeJA treatment (Figure 2D,E). In brief, the above results indicate that *PbeSTOP2* positively responded to *Vp*M and exogenous MeJA exposure.

### 3.3. PbeSTOP2 Positively Regulates Valsa Canker Resistance of Apple and Pear Fruits

**Figure 3:**
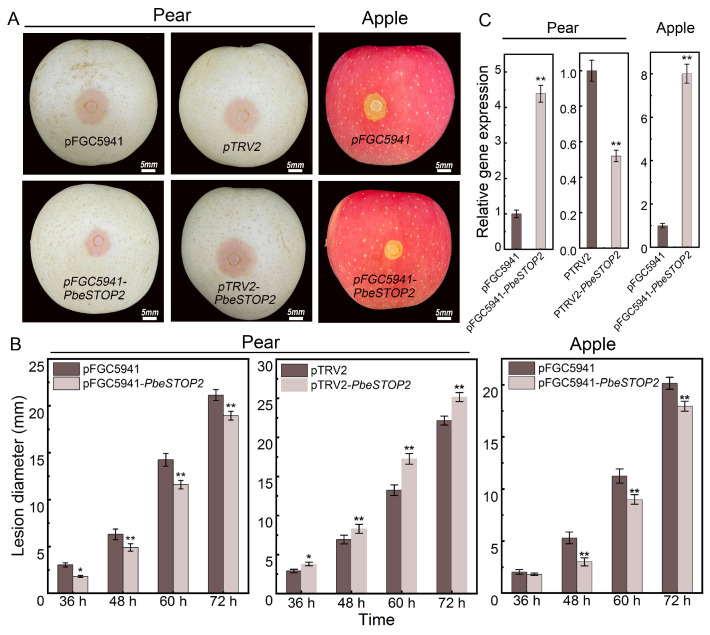
Functional characterization of *PbeSTOP2* in *Valsa* canker resistance. (**A**) Overexpression was achieved using the pFGC-5941 vector and virus-induced gene-silencing was achieved using the pTRV 2 vector. The fruits injected with empty vector pFGC-5941 or pTRV2 were used as controls. The images of the fruits were taken at 2 d post-inoculation (dpi) with *Vp*. (**B**) Lesion diameters of overexpression, silenced, and empty-vector controls infected with *Vp* on pear and apple fruits. (**C**) Expression levels of *PbeSTOP2* at the infection sites in pear and apple fruits was examined using pFGC-5941 or pTRV2 as controls. Statistical annotations: * *p* < 0.05, ** *p* < 0.01 (*t*-test); bars show mean ± SD (*n* = 5).

To investigate the role of the *PbeSTOP2* gene in *Valsa* canker resistance, we performed overexpression and virus-induced gene silencing (VIGS) of the target gene via *A*. *tumefaciens*-mediated transformation in fruits of ‘Huangguan’ pear and ‘Yanfu 6’ apple, respectively. Examination of lesions showed that transiently transformed fruits overexpressing *PbeSTOP2* had significantly decreased lesion diameters compared to controls. In addition, VIGS experiments showed that lesion diameters were significantly increased in *PbeSTOP2* silenced fruit compared to those in the control (Figure 3A,B). Overexpression and silencing of the genes at the infection site was confirmed by qRT-PCR (Figure 3C). These findings indicate that *PbeSTOP2* acts as a positive regulator of resistance against *Vp*.

### 3.4. PbeSTOP2 Overexpression Enhanced Vp Resistance of ‘Duli-G03’ Suspension Cells

**Figure 4:**
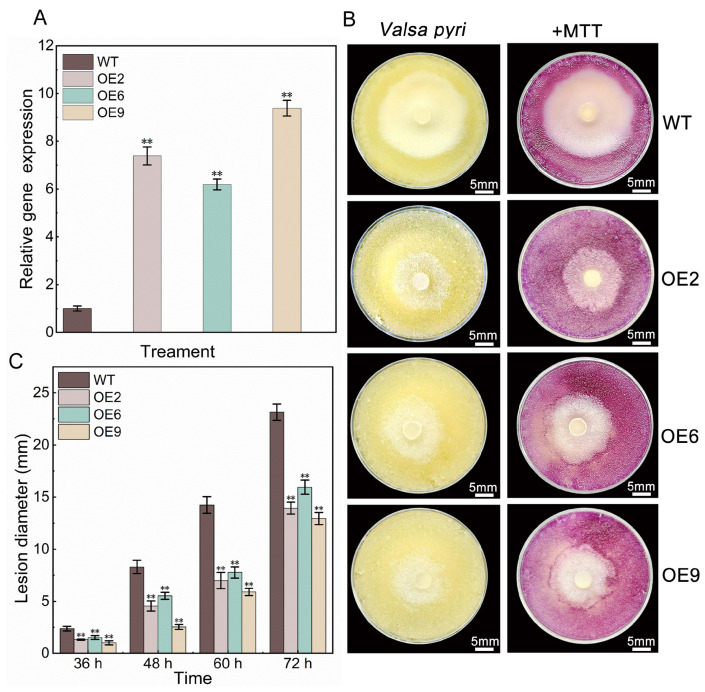
Enhanced *Vp* resistance in ‘Duli-G03’ suspension cells via *PbeSTOP2* overexpression. (**A**) Expression levels of *PbeSTOP2* in WT and overexpressed cell lines. (**B**) Colony growth phenotype at 72 h post-inoculation. +MTT: Viability assessment via methyl thiazolyl tetrazolium (MTT) staining. (**C**) Lesion metric analysis showing diameter differentials between different genotypes. Statistical annotations: ** *p* < 0.01 (*t*-test); bars show mean ± SD *(n* = 5).

To further validate the regulatory role of *PbeSTOP2* on *Valsa* canker, the target gene was overexpressed in suspension cells of ‘Duli-G03’. Three independent overexpression lines (*PbeSTOP2*-OE2/OE6/OE9) exhibited transcript levels that were 8-, 6-, and 10-fold higher, respectively, than those in WT cells (Figure 4A). Pathogen inhibition assays revealed markedly suppressed mycelial growth of *Vp* on all overexpression lines compared to the WT cells (Figure 4B). These observations were further corroborated by MTT-based viability assays. Quantitative detections recorded the colony diameter from 13 to 16 mm on overexpressing cell lines, whereas the diameter was 23 mm on the WT cells (Figure 4C). These findings demonstrate that elevated expression of *PbeSTOP2* significantly enhances resistance to *Valsa* canker in ‘Duli-G03’ cells.

### 3.5. PbeSTOP2 Initiated Immune Responses

**Figure 5:**
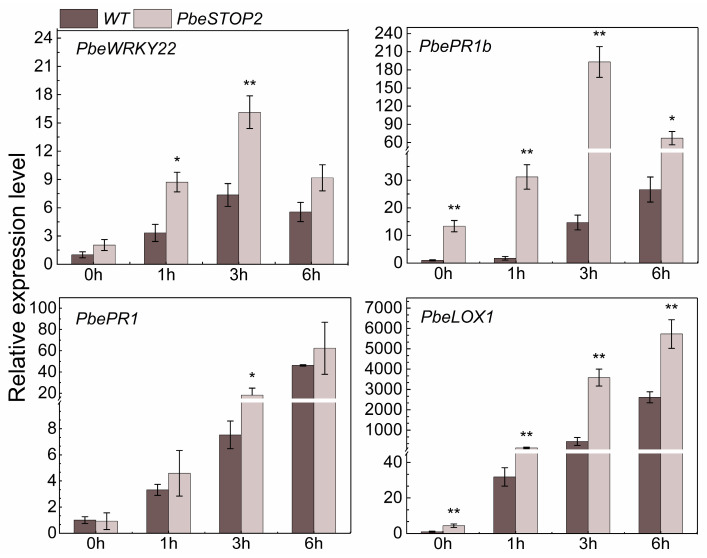
Expression levels of the immunity-related genes in WT and *PbeSTOP2*-OE9 treated with 20% *Valsa pyri* metabolites (*Vp*M). *PbeWRKY22* was related to PTI, *PbePR1* was related to SA, and *PbePR1b* and *PbeLOX1* were related to JA. Statistical annotations: * *p* < 0.05, ** *p* < 0.01 (*t*-test); bars show mean ± SD (*n* = 5).

To investigate the signaling pathway activated by *PbeSTOP2*, we analyzed the expression of four defense-related marker genes during ‘Duli-G03’ suspension cells response to *Vp*M signals (Figure 5). Compared with the WT cells, the expression of the PTI-related gene *PbeWRKY22* [31] was significantly up-regulated at 1 and 3 h. For the SA-related gene, *PbePR1* [31], the higher expression level was only observed from the overexpressing cells at 3 h compared with that from the WT cells. Strikingly, compared with the WT cells, prompt ascending of the JA-related genes *PbePR1b* and *PbeLOX1* [32] was observed from the overexpressing cells at all four time points. Therefore, the above data suggest that JA and other defense-related signals are involved in regulating the defense response of *PbeSTOP2.*

### 3.6. PbeSTOP2 Induced the Accumulation of Defense-Related Phytohormones

**Figure 6:**
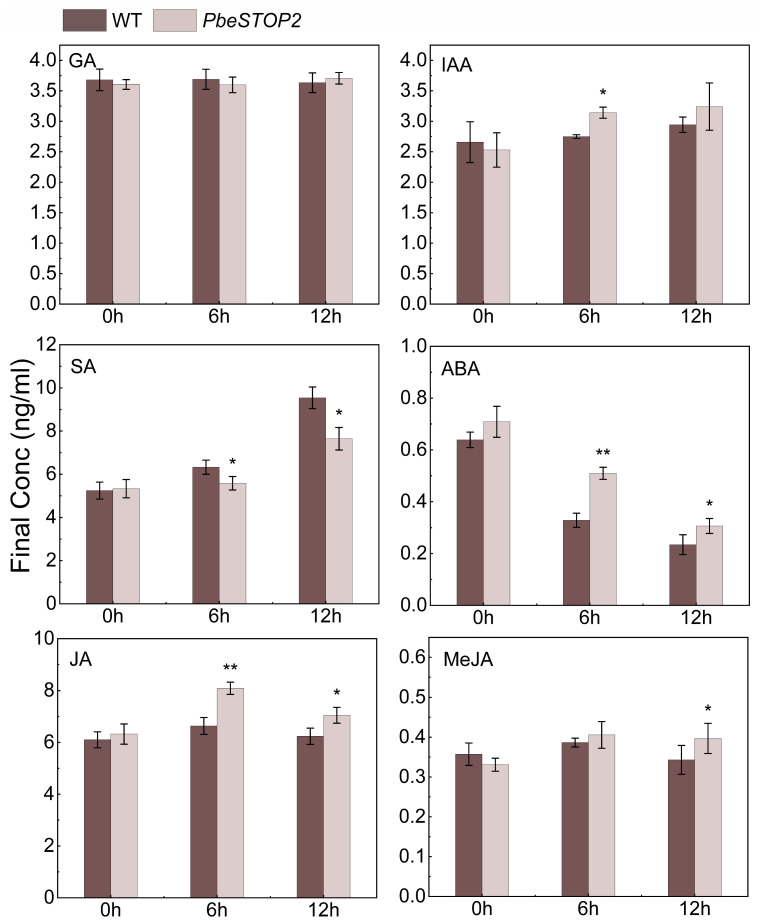
Analysis of endogenous hormones in *PbeSTOP2*-overexpressing cells and WT cells infected with 20% *Valsa pyri* metabolites (*Vp*M). Statistical annotations: * *p* < 0.05, ** *p* < 0.01 (*t*-test); bars show mean ± SD (*n* = 5).

To elucidate the function of phytohormones in the *Valsa* canker resistance mediated by *PbeSTOP2*, we examined the content of multiple hormones in overexpression and WT cells (Figure 6). For the Gibberellin (GA) levels, there was no obvious differentiation between the overexpression and WT cells. In addition, the increase in auxin (IAA) levels was observed in the overexpressing cells only at 6 h compared with the control. The lower levels of SA were discovered from the overexpressing cells than from the WT cells at 6 h and 12 h, whereas higher levels of abscisic acid (ABA) were detected. Notably, compared to WT cells, only JA levels were significantly elevated in overexpression cells at 6 h, whereas both JA and MeJA showed marked increases by 12 h. Thus, these data revealed the crucial roles of JA signals on *PbeSTOP2*-regulated *Valsa* canker resistance.

### 3.7. PbeSTOP2 Enhanced VpM Sensitivity and ROS Bursting of ‘Duli-G03’ Suspension Cells

**Figure 7:**
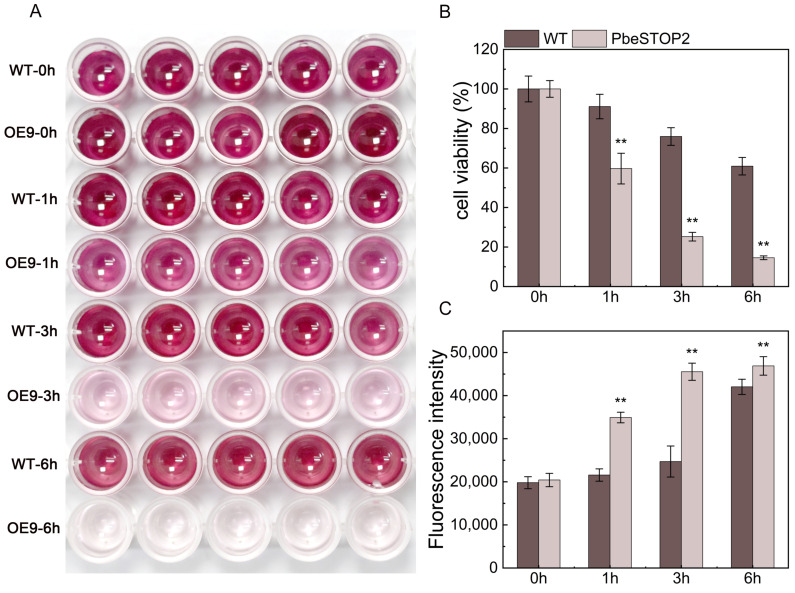
Survival and ROS analysis of ‘Duli-G03’ WT and *PbeSTOP2*-OE9-overexpressing cells after 20% *Valsa pyri metabolites* (*Vp*M) treatment. (**A**) The WT and overexpressing cells (OE9) under 20% *Vp*M treatment were stained with methyl thiazolyl tetrazolium (MTT). (**B**) Cell viability of the WT and *PbeSTOP2*-overexpressing cells treated with 20% *Vp*M. (**C**) Changes in the active oxygen content of the WT and *PbeSTOP2*-overexpressing cells after *Vp*M challenge. Statistical annotations: ** *p* < 0.01 (*t*-test); bars show mean ± SD (*n* = 5).

Cell viability and ROS accumulation were also investigated to clarify the potential mechanisms of *PbeSTOP2*-conferred *Valsa* canker resistance. The cell viability of *PbeSTOP2* was significantly decreased compared with the WT cells at 1, 3, and 6 h of *Vp*M exposure (Figure 7A,B). In contrast, ROS quantitative analysis showed that the relative fluorescence ratio in *PbeSTOP2*-OE9 was significantly higher than in the WT cells at all time points (Figure 7C). Thus, these data also imply that cell viability and ROS accumulation are involved in *PbeSTOP2*-mediated *Valsa* canker resistance.

## 4. Discussion

As a result of evolution, plants have developed a series of stress-responsive protective mechanisms by regulating intracellular gene ex patterns pression, and an increasing number of TFs have been found to play key roles in responding to stress. In this study, we found that *PbeSTOP2* was a typical C2H2-type TF, and the transcription of *PbeSTOP2* was robustly induced by *Vp*M and MeJA. Importantly, overexpression of *PbeSTOP2* significantly enhanced the *Valsa* canker resistance of apple and pear fruits and ‘Duli-G03’ cells, and activation of the JA-related pathway was critical to the enhanced resistance.

In organisms, TFs directly or indirectly recognize and bind to the promoter region of target genes, activate the RNA polymerase activity of the dynamic transcription complex, and thus accomplish the regulation of gene expression [12,33]. Upon recognition of stress-associated cis-elements, transcription factors can respond to stress on C2H2-type gene regulation [34]. For instance, LOS2 (a bi-functional enolase) represses *STZ/ZAT10* expression by binding to its promoter [35]. The cold stress C-repeat binding factor (CBF3) positively regulates gene *ZAT10* expression through a DRE *cis*-element [36]. The distribution of multiple stress-related *cis*-elements in the *PbeSTOP2* promoter region suggests that the regulation and expression of the target gene may be synergistically regulated by different hormones. In our study, we confirmed that the expression level of *PbeSTOP2* was strongly induced by exogenous JA. These results implied that the resistance of *PbeSTOP2* to *Valsa* canker is likely to depend on the activation of JA-related pathways.

Plants sensing pathogen signals stimulate their own PTI response, immediately followed by the initiation of nonspecific basal defense responses, manifested by the activation of the mitogen-activated protein kinase (MAPK) cascade, rapid deposition of callose-rich cell wall enforcements, bursts of ROS, etc. [37]. ROS, as part of the cellular signaling network, can be integrated with other signaling pathways such as ABA, SA, and JA to defend against pathogen infestation. It has been shown that ROS plays a major role in both PTI and ETI responses [38,39]. ROS production by the NADPH oxidase RBOHD is a critical early signal linking PRR and NLR-mediated immunity, and the receptor-like cytoplasmic kinase BIK1 is integral to this process [40]. In *Arabidopsis*, D36E (avrRpt2)-treated co-receptor mutants showed differential expression of genes regulated by *BIK1*, including many PTI immune pathway marker genes, such as *WRKY22/29* and *FRK1* [39]. However, when the concentration of ROS is too high, it can also lead to programmed cell death (PCD) and act as a layer 1 defense during a pathogen attack [41]. Signaling molecules that promote death through autophagy during pathogen-induced hypersensitivity response programmed cell death (HR-PCD) restrict cell death to the infected tissue site, effectively controlling the spread of HR to healthy tissues [42,43]. Following the silencing of Beclin1, a homolog of the yeast autophagy-related gene *ATG6/Beclin1*, HR-PCD triggered by tobacco mosaic virus (TMV) infection in tobacco was uncontrolled and spread to uninfected tissues [44]. In this study, a significant ROS burst was observed in *PbeSTOP2*-overexpressing cells after *Vp*M infection compared with the WT cells, and the PTI-related gene *PbeWRKY22* was up-regulated. Nevertheless, *PbeSTOP2*-overexpressing cells infected by *Vp*M exhibited lower cell viability than the WT cells. We suggest that ROS production during *PbeSTOP2*-mediated immunization may act as a signaling molecule to initiate downstream molecular cascade reactions, while the burst of ROS caused a decrease in *PbeSTOP2*-overexpressing cell viability.

The response of plants to pathogen attacks is the result of a series of changes at the cellular level, which are mediated by hormone signals, such as SA, JA, ET, IAA, ABA, etc. [45]. Studies have shown that there is an overlap between different hormone signaling pathways, resulting in tightly regulated plant resistance mediated by these signaling pathways [46]. It is generally accepted that SA is involved in defense against biotrophic pathogens, whereas JA and ET contribute to defense against necrotrophic pathogens [47]. The effect of SA on the JA pathway can be antagonistic, synergistic, or neutral [48,49]. Previous studies have identified several members that play a role in JA-SA antagonism, including *MYC2*, *PDF1.2*, *NPR1*, *WRKY62, GRX480*, *JAZs*, etc. [50,51]. A recent gene-wide transcriptome study revealed a wide range of synergistic effects of JA-SA in *Arabidopsis* [52]. In this study, the expression of JA signaling-related genes *PbeLOX1* and *PbePR1b* was strongly induced in the *PbeSTOP2*-overexpressing cell after *Vp* infection compared with the WT cells. Furthermore, the endogenous JA concentration was significantly higher in the transgenic cells than in the WT cells after pathogen inoculation. Differently, although the expression of the SA-related gene *PbePR1* was up-regulated in *PbeSTOP2*-overexpressing cells, the endogenous SA content was lower than that of the WT cells. Therefore, we suggest that the disease resistance conferred by *PbeSTOP2* depends on the JA signaling pathway but not the SA signaling pathway.

Furthermore, JA signaling engages in complex interactions with other phytohormones to coordinate plant immunity. Among them, IAA positively regulates necrotrophic pathogens; for instance, in *Arabidopsis*, IAA-deficient mutants are more sensitive to necrotrophic pathogens than WT [53]. Impairment of the auxin-activated SCF (Skp1–Cullin–F-box) ubiquitination pathway in *Arabidopsis* mutants *axr1*, *axr2*, and *axr6* confers increased susceptibility to *Plectosphaerella cucumerina* and *Botrytis cinerea* [54]. Following *Vm* infection, genes associated with IAA and ABA signaling pathways exhibit downregulated expression in susceptible apples [55]. However, the role of ABA in plant disease resistance is complex and appears to vary in different plant-pathogen interactions [56]. In addition, ABA has multifaceted roles in different stages of plant defense. In the initial stage, ABA resists fungal invasion by mediating stomatal closure [57]. In the second stage, it promotes callus deposition in response to fungal infection [58]. Finally, ABA interacts with SA-, JA- and ET-dependent defense pathways. For example, ABA regulates JA-dependent resistance by inhibiting the JA response that is synergistically regulated by ET [59]. but promotes the JA branch that is antagonistic to ET [60]. In the present study, upregulation of IAA hormone concentration in *PbeSTOP2*-overexpressing suspension cells after infestation with *Vp* implies that IAA is presumably involved in *PbeSTOP2*-mediated disease resistance. Unusually, the endogenous ABA concentration was significantly higher in *PbeSTOP2*-overexpressing suspension cells than in WT cells after *Vp* infection. Therefore, the mechanism of ABA in the regulation of *Valsa* canker resistance by *PbeSTOP2* requires further validation.

## 5. Conclusions

In summary, *PbeSTOP2*, a member of the C2H2-type zinc finger protein family, functions as a positive regulator of resistance to *Valsa* canker in both ‘Duli-G03’ cells and fruits of pear and apple. The signal pathways associated with ROS, cell death, and JA are probably involved in the immune response regulated by *PbeSTOP2*. Moreover, the results of expressional assay and hormone determination suggest that JA signals take crucial roles in the launching of *Valsa* canker resistance caused by *PbeSTOP2*. Therefore, the results of this study provide new insight into resistance breeding and further comprehensive prevention and control for the occurrence of *Valsa* canker.

## Figures and Tables

**Table 1:** Primers for PCR and quantitative real-time PCR.

Application	Gene Name	Gene Accession	Forward Primer	Reverse Prime
PCR	PbeSTOP2	Chr3.g19672	GCGGCGCGCCATGGTTTCTGCGGCCCCATCTGATT	GCCCTAGGTCATCCCAATTGCATTTGCGATCCA
qRT-PCR	PbeSTOP2	Chr3.g19672	CGTCACGGCTTCTTCGATGA	TATCAGCGACGCCAAGGTTT
Reference genes	β-Tubulinc	Chr3.g20215	TTCAGATACTGTTGTGGAGCCTTAC	AGTAACTCCAGACATTGTTGCAGAG
VIGS	PbeSTOP2	Chr3.g19672	CAACGCGTGTTTCTGCGGCCCCATCTGATTGT	GACCCCCGCATCCAAGAAGCCGTGACCATGAT
PTI	PbeWRK22	Chr7.g33338	CATATCCAAGGGGATATTACAGATG	GTGACTATAAAAATATTCGGGTCGG
JA	PbePR1b	Chr5.g08127	GACACACCCCAAGACTACCTCAAG	GTCACCAGTGCTCATGGCAAG
JA	PbeLOX1	Chr4.g40498	GCTTATGTGGCTGTAAATGACTCTG	GAGGATGCAGAAGTTTGTAAATTGG
SA	PbeRP1	Chr5.g08131	AATCTTGTTCATTTTGGTGGGCC	AACAACCTGAGTATAATGCCCACAC

## Data Availability

The original contributions presented in this study are included in the article/Supplementary Materials. Further inquiries can be directed to the corresponding authors.

## Supplementary Materials

The following supporting information can be downloaded at: https://www.mdpi.com/article/10.3390/cimb48010014/s1.
