# Supplementary figures and images for: Jasmonic Acid Signals Involved in Valsa Canker Resistance Caused by C2H2-Type Transcription Factor PbeSTOP2 in Pyrus betulifolia
Source: Curr Issues Mol Biol. 2025 Dec 23;48(1):14. doi: 10.3390/cimb48010014 (PMC12839958; doi:10.3390/cimb48010014)

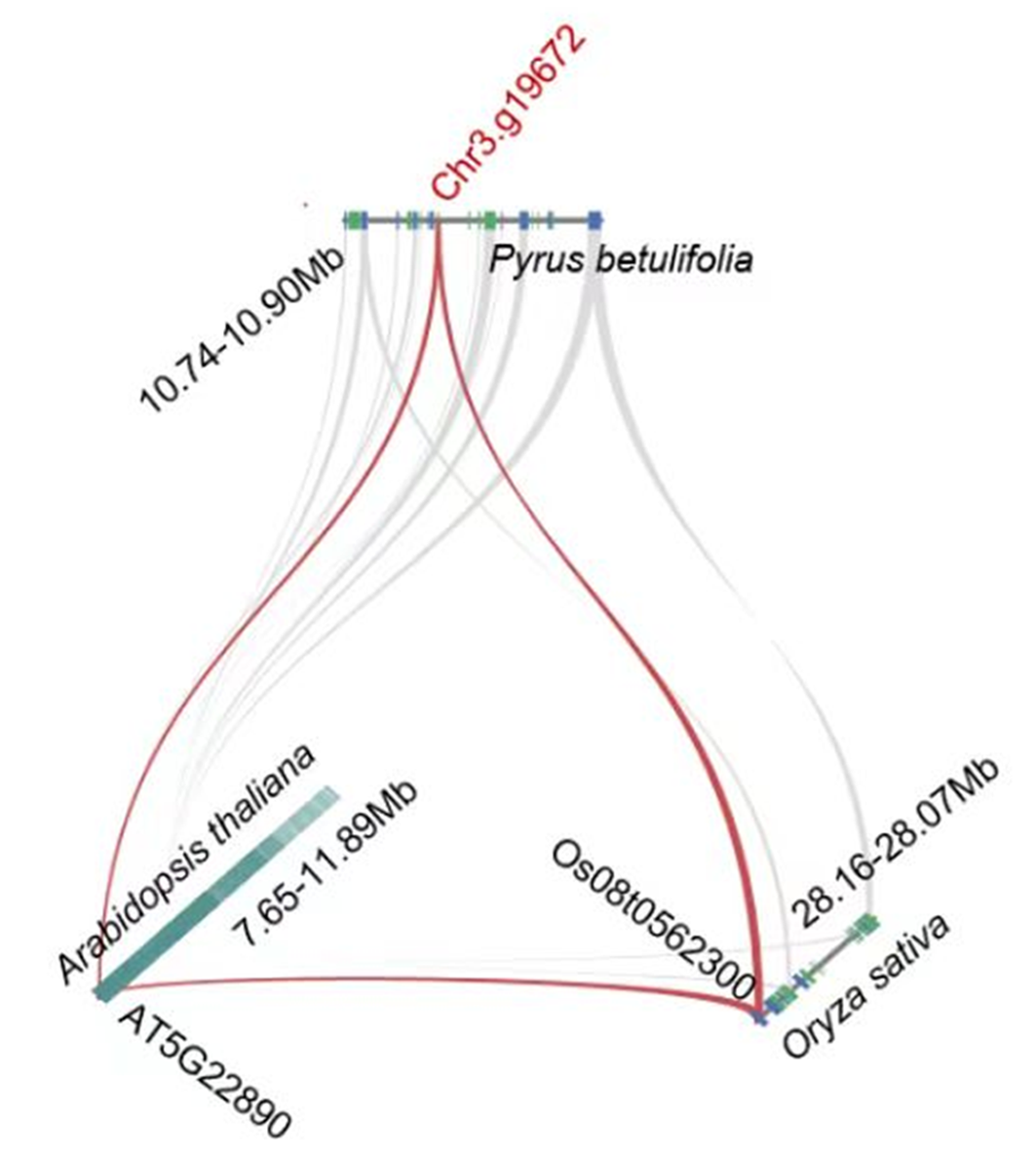

Supplement: Supplementary file 1 [file cimb-48-00014-s001.zip › Figure. S 1.png]
